# Development of Gold Nanoparticle-Based SERS Substrates on TiO_2_-Coating to Reduce the Coffee Ring Effect

**DOI:** 10.3390/nano12050860

**Published:** 2022-03-03

**Authors:** René Breuch, Daniel Klein, Cassandra Moers, Eleni Siefke, Claudia Wickleder, Peter Kaul

**Affiliations:** 1 Institute of Safety and Security Research, University of Applied Sciences Bonn-Rhein-Sieg, von-Liebig-Str. 20, 53359 Rheinbach, Germany; daniel.klein@h-brs.de (D.K.); cassandra.moers@h-brs.de (C.M.); eleni.siefke@h-brs.de (E.S.); 2 Inorganic Chemistry, Department Chemie and Biologie, Cµ—Center for Micro- and Nanochemistry and (Bio)Technology, Faculty of Science and Technology, University of Siegen, Adolf-Reichwein-Str., 57068 Siegen, Germany; wickleder@chemie.uni-siegen.de

**Keywords:** Raman spectroscopy, AuNPs, TiO_2_-coatings, coffee ring effect, SERS

## Abstract

Hydrophilic surface-enhanced Raman spectroscopy (SERS) substrates were prepared by a combination of TiO_2_-coatings of aluminium plates through a direct titanium tetraisopropoxide (TTIP) coating and drop coated by synthesised gold nanoparticles (AuNPs). Differences between the wettability of the untreated substrates, the slowly dried Ti(OH)_4_ substrates and calcinated as well as plasma treated TiO_2_ substrates were analysed by water contact angle (WCA) measurements. The hydrophilic behaviour of the developed substrates helped to improve the distribution of the AuNPs, which reflects in overall higher lateral SERS enhancement. Surface enhancement of the substrates was tested with target molecule rhodamine 6G (R6G) and a fibre-coupled 638 nm Raman spectrometer. Additionally, the morphology of the substrates was characterised using scanning electron microscopy (SEM) and Raman microscopy. The studies showed a reduced influence of the coffee ring effect on the particle distribution, resulting in a more broadly distributed edge region, which increased the spatial reproducibility of the measured SERS signal in the surface-enhanced Raman mapping measurements on mm scale.

## 1. Introduction

Surface-enhanced Raman spectroscopy (SERS) is a special variant of Raman spectroscopy that is based on the enhancement of Raman scattered light, which can provide detailed information about molecular structures of a high variety of samples [1,2]. The surface enhancement originates from two main mechanisms, which amplify the Raman signal by an electromagnetic (EE) and chemical (CE) enhancement in the presence of nanostructures, mostly metallic nanostructures like silver or gold. Thereby CE originates from a charge transfer between a nanostructure like spherical nanoparticle and an adsorbing molecule. The charge transfer leads to an altered electronic structure of the molecule or the metal-adsorbate complex, which can lead to a higher Raman excitation probability. The EE, on the other hand, is caused by the formation of a locally enhanced electromagnetic field at for example plasmonic-metallic nanostructures. If such structures are present in near-surface regions, the electromagnetic field from the plasmons that develops by light interaction, leads to an amplification of both the incident excitation light and also of the emitted Raman scattered light [3,4]. Over the years, several methods for the fabrication of SERS-substrates found their way on to the commercial market and can now be bought and used in different analyses [5,6,7,8].

The nanostructures, which can be used for SERS, vary from surface modification [9], 3D-structures [10] to several forms of nanoparticles. In literature, a high variety of morphologies of nanoparticles are used for SERS. Nanodots [11], nanotriangles [12], nanocubes [13], nanostars [14], nanopillars [15], nanorods [16], nanopopcorns [17], nanosponges [18], nanomushrooms [19], nano-islands [20], bioinspired micropatterned Au-Areoles [21] and spherical nanoparticles [8,22,23,24,25] are often used in this context. A very common synthesis described by Turkovich et al., which is based on the reduction of chloroauric acid with sodium citrate and was modified several times over the years [26,27,28,29]. Beside the morphology, the scale of the nanoparticles has a big influence on the enhancement, therefore controlling the diameter of e.g. spherical nanoparticles during the synthesis is very important [24,25]. Due to the small scale of the nanoparticles, they are able to form surface plasmon resonance in the visible electromagnetic range. The connection between the nanoparticle scale (diameter) and the surface plasmon resonance can be used to estimate the mean particle diameter [27,30,31].

Beside the synthesis of nanoparticles, the uniform application and the immobilisation of the nanoparticles onto surfaces are major tasks [32]. A common problem in the processing of nanoparticle suspensions is the non-uniform particle distribution after drying on a surface. Concerning the starting material components used in the synthesis of nanoparticles, the suspensions are mostly water-based. When nanoparticles are applied to a surface by drop coating, the so-called coffee ring effect (CRE) occurs [33]. This phenomenon was found to be caused by capillary flow during the drying of the suspension in studies by Deegan et al. This flow transports the particles to the edge of the fluid droplet [34] due to the faster solvent evaporation at the edge of the droplet [35]. The effect can be influenced by various parameters, such as surface tension, surface properties or drying conditions [36,37]. To reduce the influence of the CRE, different techniques can be found in literature, like modifying the liquid properties by changing the viscosity, the boiling point (vapour pressure) [36] or the drying environment [35]. In addition, the use of surface acoustic waves are studied to reduce the influence of the CRE [36]. Moreover, the surface properties, like porosity and wettability can also be altered to reduce the influence of the CRE [36]. Another method to control the CRE is changing the surface morphology by applying nanofibers [38]. A different possible way is to modify the wettability by chemical functionalisation of the surface for example via linker agents, like 3-aminopropyltriethoxysilane (APTES) [39], (3-mercaptopropyl)trimethoxysilane (MPMS) [23] or 3-mercaptophenylboronic acid [40]. Some of these organic linker agents can capture noble metal nanoparticles and, thus, help to form more uniform particle distributions. As a drawback, these organosilanes can result in a background spectrum [40,41] and interfere with a target analyte spectrum, especially due to the organic functional groups of the organosilanes.

An inorganic alternative to influence the surface wettability is the modification with metal oxide coatings like TiO_2_ [32,42,43]. TiO_2_ can be synthesised in different modifications and morphologies [32,44,45] and can be used for coating aluminium [46] and changing surface wettabilities [32]. For a previous cleaning and activation of aluminium surfaces, plasma treatments can be used [47].

Further, TiO_2_-nanostructures were also investigated concerning their ability to enhance Raman spectra. Musumeci et al. showed in a communication letter in 2009 that charge transfer complexes of different bioorganic substances containing TiO_2_-nanoparticles were able to cause an enhancement in the Raman signal [48], which was also reported by Yang et al. in 2008 [49] and theoretically described in 1983 by Ueba [50]. Also other semiconductors show such a behaviour, like zinc oxide [51], copper oxide [52] molybdenum oxide [53], molybdenum disulfide [54] or iron oxide [55]. For TiO_2_ coatings on aluminium, different methods were investigated over the years, like brush plating [56], plasma spray coating [57], micro-arc oxidation [58], vacuum dip coating [59] spin or dip coating of sol gel precursor like titanium tetraisopropoxide (TTIP) [60,61]. TTIP is an often used precursor molecule for developing TiO_2_-coatings and -nanoparticles [61,62] and the decomposition of TTIP to TiO_2_ was studied several times [63,64,65]. The conversion of TTIP through Ti(OH)_4_ to TiO_2_ proceeds via hydroxylation and condensation reactions, whereas the condensation reactions can be divided into alcoxolations, oxolations and olations [65].

In addition to SERS applications, TiO_2_ is also known for its photocatalytic activity, for instance as a photocatalyst in chemical industry [66,67,68], or also to inactivate bacterial cells [44]. This photocatalytic activity is suitable for cleaning the substrate after application, so the substrates can be used multiple times. This is very remarkable since most SERS substrates are single-use consumables [69,70,71] and in literature only a few other options are known to clean a SERS substrate, like plasma cleaning [72] or electrochemical cleaning [73]. To further increase the Raman enhancement for SERS application, combinations of metal oxides and noble metal nanostructures were also investigated over the years, for example tin oxide with gold nanoparticles (AuNPs) [74], tin oxide with different metal nanoparticles [75], AuNPs with SnO_2_-nano wires [76], iron oxide with AuNPs [41], silicon oxide with AuNPs [77], zinc oxide with silver nanostructures and graphene oxide [78] or TiO_2_ with silver nanoparticles (AgNPs) [79].

Ambroziak et al. showed an Ag/TiO_2_ SERS substrate in their publication in 2020, where a TiO_2_ coating results in a more uniform particle distribution and a reduced CRE for their SERS substrates by decreasing the contact angle through a TiO_2_ coating, before subsequently covering the TiO_2_ coating with SERS-active cubic AgNPs. They showed a more uniform Raman enhancement on 60 µm by 60 µm scale [32]. Based on the aim of this work is the application of a plasma-treated TiO_2_-coated aluminium substrate to increase the wettability of the coating before applying an AuNP-suspension to create SERS substrates with an active surface in mm scale, which have nearly no intrinsic spectra and are not limited to small laser powers like paper-based SERS-substrates.

## 2. Materials and Methods

### 2.1. Gold Nanoparticle Synthesis

The gold nanoparticle synthesis was carried out following a modified synthesis route of Turkevich et al. [28] via a reduction of trisodium citrate. Therefore, 32 mg of trisodium citrate dihydrate (Alfa Aesar, Haverhill, MA, USA) and 30 mL of deionised (DI) water were mixed in a three-neck flask and heated to boiling with continuous stirring (700 RPM) under reflux cooling. A chloroauric acid solution consisting of 10 mL of 0.36 mg/mL hydrogen tetrachloroaurate (Alfa Aesar, Haverhill, MA, USA) was prepared and completely added when the citrate solution reached the boiling point. The mixture was then boiled for 20 min. The dispersion was afterwards slowly cooled to room temperature. Subsequently, 1 ml of the resulting suspension was diluted with 2 ml DI water and analysed in a range from 300 nm to 700 nm by UV-Vis absorption spectroscopy with an 6850 UV/Vis spectrophotometer (Jenway, Staffordshire, UK) in a polystyrene cuvette.

### 2.2. Fabrication of the SERS-Substrates

Starting with the alkaline surface pre-treatment from Batan et al. [80], the aluminium plates were immersed in a 0.6 M sodium hydroxide solution (NaOH) for 6 s at approximately 70 °C for cleaning and surface activation, then the substrates were rinsed with DI water and subsequently air-dried [80].

Afterwards a plasma cleaner (Zepto One, Diener electronics, Ebhausen, Germany) was used for further surface cleaning by means of low-pressure plasma. The aluminium plates were exposed to the plasma for 30 min directly after the alkaline pre-treatment with the aim of removing possible organic residues and other contamination.

For the coating of the aluminium substrates, solutions with different TTIP (titanium isopropoxide, ≥95%, Alfa Aesar, Haverhill, MA, USA) volume concentrations were prepared. The TTIP concentrations 1%, 2%, 3%, 4%, 5% and 10% were prepared in isopropanol (isopropanol, ≥99.5%, Merck, Darmstadt, Germany). The cleaned aluminium substrates were rinsed with 2 µL of the TTIP solutions. Afterwards, the substrates were either dried under ambient air for 24 h or calcinated and sintered at 600 °C for 5 h in a muffle furnaces L-09/13 (Nabertherm, Lilienthal, Germany).

The substrates without heat treatment were directly coated with AuNPs after a drying time of 24 h. The calcinated SERS-substrates were again cleaned by the plasma cleaner for 30 min, to remove residues from the calcination process, and to activate the TiO_2_ surface before being coated with the synthesised AuNPs. For both types of substrates, 2 µL of the gold nanoparticle suspension were applied onto the clean TiO_2_ coatings by a microliter pipette and dried under ambient air.

### 2.3. SEM Analysis

For the characterisation of the samples after the fabrication a SEM microscope (JSM-7200F, JEOL, Akishima, Japan) was used. The images were taken with a secondary electron detector at a primary beam energy of 10 kV and 15 kV under a high vacuum of 10^−^^4^ Pa. The SEM images were obtained at a long scan acquisition time, which resolves in a freeze time of 76 s per image.

### 2.4. Water Contact Angle Measurements 

Water contact angle (WCA) measurements were carried out using a VHX-600 microscope with the VH-Z20R objective (Keyence, Osaka, Japan). Each type of treatment as well as a blank aluminium slide were measured. Therefore, 5 µL of water were pipetted onto the surface and measured by the microscope in a 90° angle. Five different measurements were made on each of the three substrates per treatment.

### 2.5. Raman Analysis

In this study, a fibre-coupled Raman spectrometer QE-Pro (Ocean Insight, Orlando, FL, USA) with a charge-coupled device (CCD) detector was used. The SERS-substrate samples were placed on a motorised XY-stage LTS 150 (Thorlabs, Newton, NJ, USA) and focused with a fibre-coupled probe (InPhotonics, Norwood, MA, USA) with a spot size of approximately 160 µm in diameter. The measurements were performed with a 638 nm laser and 35 mW (InPhotonics, Norwood, MA, USA). A LabVIEW software (National Instruments, Austin, TX, USA) carried out the controlling of the spectrometer and XY-stage as well as the data acquisition.

Rhodamine 6G (R6G) was used as a target molecule like in several other publications in the field of SERS [7,33]. Therefore, 2 µL of a 10^−6^ mol/L R6G were applied by a microliter pipette onto the SERS substrates, resulting in the application of 0.96 ng R6G onto the substrates.

All measurements were collected with an integration time of one second. For small area mappings, at each measurement position of a 3 by 3 raster with 0.3 mm distance between each point, 10 spectra were collected.

For large-area mappings, the plates were measured on a 20 by 20 grid at 0.2 by 0.2 mm intervals. The resulting 4000 measurement positions are each composed of the mean values of 10 individual spectra.

Additionally, to investigate the TiO_2_ layer a Raman microscope (Senterra, Bruker, Billerica, MA, USA) was used to focus on the thin layer. For the investigation, a 100× long range objective with a numerical aperture of 0.8 (LMPlanFL N, Olympus, Shinjuku, Japan) and a 488 nm laser with 40 mW were used. The measurements were done with 1 s integration time, 5 co-additions, a spot diameter of ~5 µm and a spectral resolution of 3~5 cm^−^^1^.

## 3. Results and Discussion

### 3.1. Synthesis of Gold Nanoparticles

**Figure 1:**
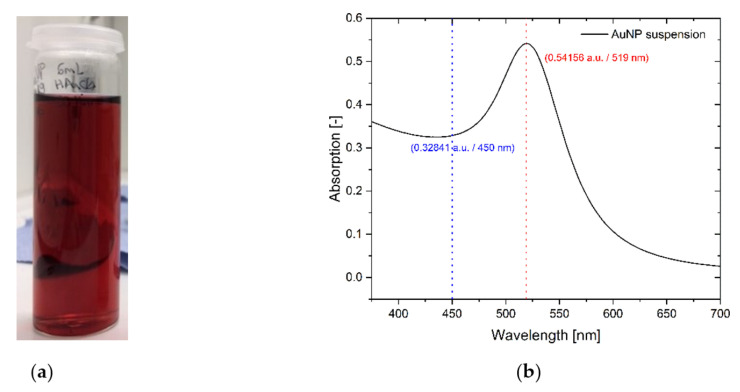
Synthesised gold-nanoparticles (AuNPs), (**a**) image of the AuNPs suspension. (**b**) UV-Vis absorption spectrum of a AuNPs suspension with a maximum at 519 nm (red dotted line) and the reference intensity at 450 nm (blue dotted line).

The synthesis of gold nanoparticles was done as described in the experimental section. During the boiling process, the initially yellowish solution of the chloroauric acid changes colour first to light red then to dark red as the nanoparticles are formed. The intense red colour caused by the plasmonic resonance band can be seen in Figure 1a and the corresponding UV-Vis spectra in Figure 1b.

The position of the plasmonic resonance band and the intensity allow an approximation for the mean particle size. Haiss et al. [31] showed the connection between the absorption maxima of the Vis spectra and the particle size of spherical AuNPs in their work. Following their calculations and using the supplementary (SI) information from Haiss et al., as well as the maxima and a reference location at 450 nm from the UV-Vis absorption spectra from Figure 1, it is possible to estimate the mean particle size. The maximum was located at 519 nm with an absorption of 0.54156 and the reference location at 450 nm with an absorption of 0.32841. Resulting in a quotient of 1.6490, which correspond to an approximately mean particle size of 16 nm [31]. Comparable results for the particle size can be found by comparing the position of absorption maxima with the results of He et al. [30] Additionally a high resolution SEM images of the AuNPs on the final SERS substrate can be seen in the Supplementary Materials (Figure S1).

### 3.2. Fabrication of AuNPs-TiO_2_-SERS Substrates

In a next step, different coating solutions of TTIP were applied on cleaned surfaces. Therefore, the concentrations of TTIP in isopropanol were varied between 1% and 10% TTIP. Afterwards, the substrates were dried under ambient air for 24 h to achieve a slow conversion by hydrolysis through moisture towards Ti(OH)_4_. Since the hydrolysis rates are dependent on the accessible water content [81], the reaction proceeds slowly. During the drying of the coating, the formation of a thin white solid film was noticed on the substrates. This thin film leads to the conclusion that despite the low temperature, hydrolysis reactions had taken place, resulting in the formation of Ti(OH)_4_. Condensation reactions like alcoxolation of the remaining TTIP or oxolation of Ti(OH)_4_ for the formation of TiO_2_ are described to take place at higher temperature [32,43,63,64], but also room temperature formations of TiO_2_ were reported in literature [81,82,83] and, therefore, could also take place.

**Figure 2:**
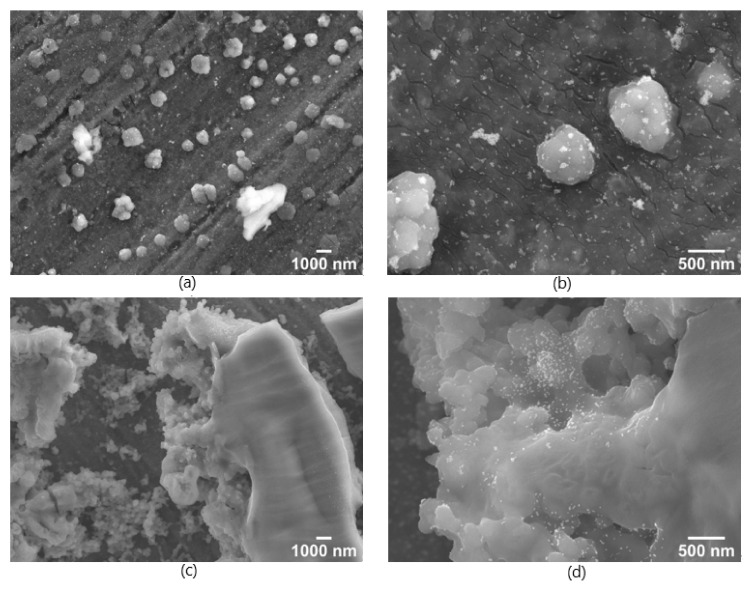
SEM-images of SERS-substrates coated with a solution of 1% (**a**,**b**) and 10% (**c**,**d**) TTIP and dried for 24 h under ambient conditions. Afterwards they were coated with AuNPs. Left side shows a 5000× magnification (**a**,**c**) and the right side a 25,000× magnification (**b**,**d**) with a working distance (WD) 4 mm and 10 kV acceleration voltage.

After the substrates were dried for 24 h, the AuNPs were applied by 2 µL drop coating, the substrates were dried again, and afterwards they were analysed by SEM. In Figure 2 the surfaces with the lowest (1%) (a,b) and the highest (10%) (c,d) TTIP concentrations are displayed.

The concentrations of the TTIP solutions have an influence on the morphological appearance of the resulting Ti(OH)_4_ coatings. At 1%, a thin layer of TTIP is covering the aluminium with several thin cracks and bigger particles of approx. 1 µm are observed. At 10% TTIP solution bigger plate-like areas of the TTIP/Ti(OH)_4_ were formed, which cover the surface and form a rough surface morphology on a micrometre scale.

**Figure 3:**
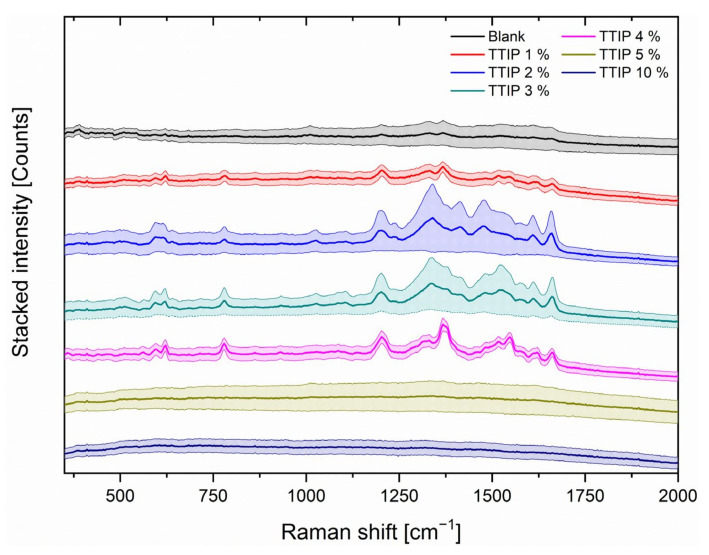
Mean Raman spectra as well as the standard deviations of a 3 by 3 mappings with the 638 nm Laser with 35 mW, 1 s integration time and 10 spectra of each mapping position of the SERS substrates with different concentration of TTIP (coloured lines) and dried under ambient air for 24 h. Additionally an untreated aluminium substrate (AuNPs, black line) is used as a reference. All substrates were coated with AuNPs suspension and 2 µL of rhodamine 6G (R6G) (10^−6^ mol/L) were applied as a model analyte.

To find the best suitable concentration of the TTIP solution for the coating, also small area Raman mappings as described in the experimental section were performed on the prepared SERS substrates. Figure 3 shows the stacked, not normalised, mean Raman spectra as well as the standard deviation of the mappings of R6G (10^−^^6^ mol/L) on the different SERS substrates from the TTIP concentration of 1%, 2%, 3%, 4%, 5% and 10% as well as an untreated aluminium substrate also covered with AuNPs.

**Figure 4:**
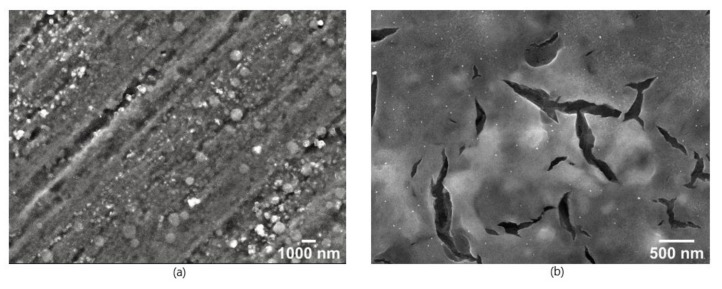
SEM images from a SERS substrate covered with a 4% TTIP solution, dried for 24 h under ambient air. Afterwards it was coated with 2 µL of the AuNPs suspension. (**a**) 5000× magnification, (**b**) 25,000× magnification with a WD 4 mm and 10 kV acceleration voltage.

The results of Figure 3 show that the coating with the TTIP concentration of 4% resulted in the most intense spectra compared to e.g. the spectra of the TTIP concentration of 1% and the coating showed the lowest variance within the spectra compared to the spectra of the other TTIP concentrations. The more favourable spectra of the substrates with 4% TTIP according to the intensity and standard deviation could be attributed to the uniform distribution of the nanoparticles and the analyte. The corresponding SEM images of substrates with 4% are shown in Figure 4.

The SEM images of the coating with the 4% TTIP solution show a similar structure to the 1% coating depicted in Figure 2, where the gold nanoparticles were distributed onto the surface of the coating. In contrast to Figure 2, the nanoparticles in Figure 4 are more uniformly distributed, which can be deduced from the significantly lower number of agglomerates. This also explains the lower standard deviation of the Raman spectra of the substrate with 4% TTIP, since the better distribution allows a more position-independent enhancement of the Raman spectra. Additionally, the more uniform distribution of the AuNPs increases the overall probability of analyte molecules being close to a nanoparticle, which explains the higher intensity in the SERS spectra. Nevertheless, the coating itself shows several cracks, which may originate from the drying of the TTIP and the slow conversion to Ti(OH)_4_ and TiO_2_. Therefore, a calcination and a sintering of the substrates before applying to the AuNPs was performed to further improve the SERS substrate surface morphology.

**Figure 5:**
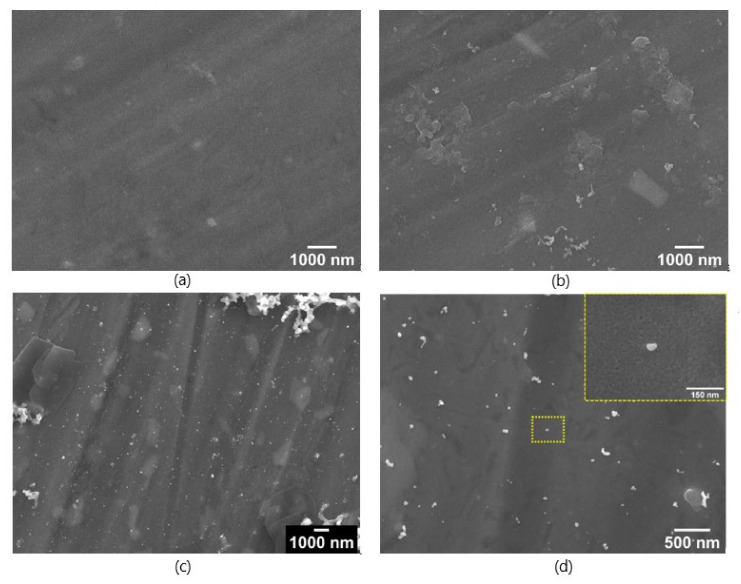
SEM image of a cleaned aluminium plate (**a**), a substrate coated with a solution of 4% TTIP calcinated to a TiO_2_-coating at 600 °C for 5 h (**b**) and coated with AuNPs. (**c**) at 5000× magnification. Additionaly more detailed SEM images from 25,000×magnification, and the inlet represents a SEM image with a 100,000× magnification are also displayed (**d**). WD 6 mm and 15 kV acceleration voltage.

To convert Ti(OH)_4_ completely to TiO_2_, by finalising the condensation reaction, plasma-cleaned aluminium plates, like Figure 5a, were heat treated in a muffle furnace at 600 °C for 5 h. Furthermore, the calcination temperature of 600 °C ensures the decomposition of the organic residues of the TTIP as well as the used solvents [63]. The plasma treatment of the surface was repeated for 30 min, to eliminate the organic residues from the heating process, displayed in Figure 5b, before the synthesised AuNPs were applied to these samples by drop coating. SEM images of the sintered and AuNP coated samples are shown in Figure 5c,d.

In contrast to the proceedings shown in Figure 1 and Figure 4, the SEM images in Figure 5 show that no cracks have occurred. The TiO_2_ coating is homogeneously distributed over the aluminium surface with a TiO_2_ nanostructured surface of the coating. This appears to consist of sintered nanograins and leads to an open porosity, which can be seen in from the inlet in Figure 5c as well in the more detail images of the surface at 25,000× magnification and 100,000× magnification in the Supplementary Materials (Figure S2). The AuNPs are distributed in smaller groups on the surface of the TiO_2_.

**Figure 6:**
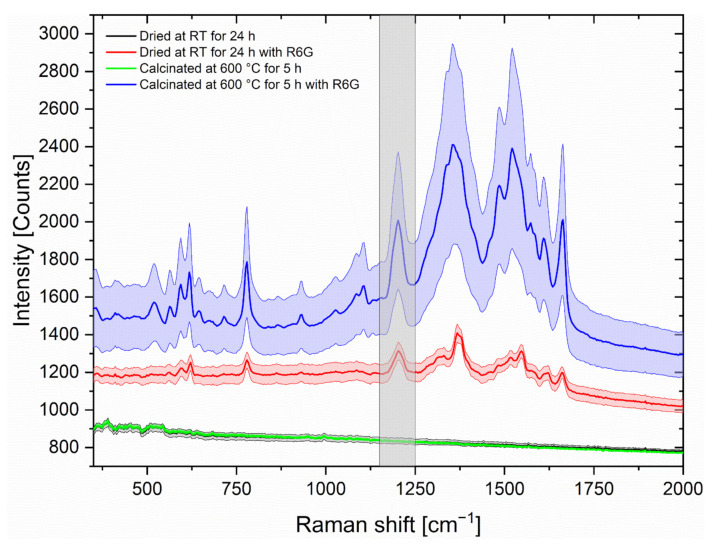
Mean SERS spectra as well as the standard deviations of a 3 to 3 mappings with the 638 nm Laser with 35 mW, 1 s integration time and 10 spectra each mapping position of the SERS-substrates with a TTIP coating dried for 24 h under ambient conditions and 5 h at 600 °C, followed by coating with the AuNPs suspension with and without R6G (conc. 10^−6^ mol/L) added.

To investigate the effect on the SERS enhancement, another small area mapping was performed after application of ~0.96 ng R6G (2 µL of 10^−6^ mol/L R6G), which can be seen in Figure 6.

The SERS spectrum of the calcinated substrate showed a significantly higher intensity than the substrate dried at room temperature, the standard deviation is also slightly higher. The background measurements on the final SERS substrates show no significant peaks without R6G, only small peaks below 700 cm^−^^1^ are noticeable.

Accordingly, the inorganic TiO_2_ background is not noticeable in the SERS measurements and therefore the SERS spectra do not need any background subtractions. Due to the high melting temperature of TiO_2_, the usable laser power is not as limited as for example for SERS substrates with cellulose or polymer as a supporting material.

**Figure 7:**
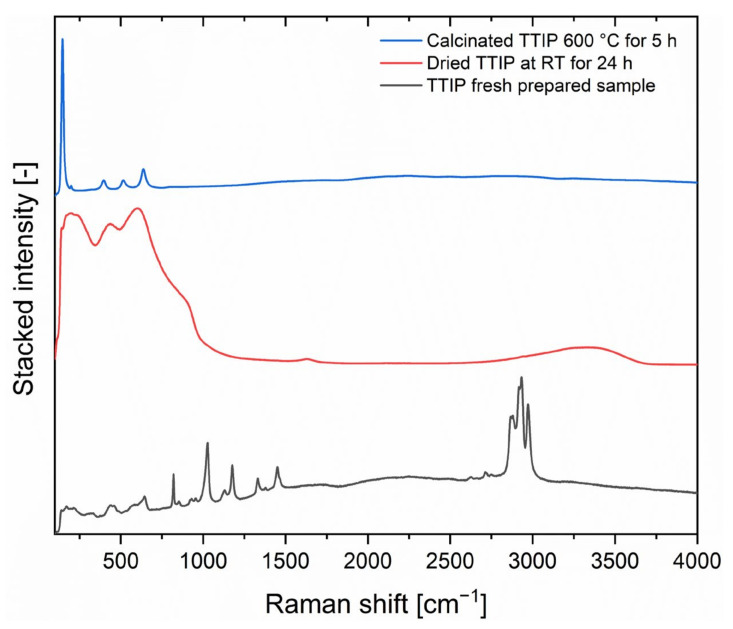
Normalised and stacked Raman microscope spectrum of the substrates with TTIP, TTIP dried for 24 h under ambient conditions and TTIP calcinated at 600 °C for 5 h. Raman spectra were collected with a 488 nm laser with 40 mW, 1 s integration time and 5 co-additions and focused with a 100× objective.

To investigate these spectral backgrounds of the SERS substrates, additional Raman measurements with a more narrow focus were performed with a Raman microscope and a shorter wavelength of 488 nm, see Figure 7.

The differences in the spectra, which are clearly visible, are attributed to the reaction of TTIP to TiO_2_ while drying. Several peaks of TTIP from 700 cm^−^^1^ to 1500 cm^−^^1^ as well as peaks from 2800 cm^−^^1^ to 3000 cm^−^^1^ disappear during drying at RT and three broad peaks at 604 cm^−^^1^, 435 cm^−^^1^ and 203 cm^−^^1^ appear as well as small peaks at 1631 cm^−^^1^ and a broad peak at 3330 cm^−^^1^.

The comparison of the TTIP substrates with the TTIP substrates dried at RT make clear, that the disappearing of the peaks from 2800 cm^−^^1^ to 3000 cm^−1^, which can be assigned to CH-vibrations [84], shows that the hydrolysis reaction had taken place and the isopropyl residue had been removed. At the same time, the small peak at 1631 cm^−1^ and the broad peak at 3330 cm^−1^ can be assigned to OH-vibration of Ti-OH groups [85]. This leads to the assumption that the coating consists of Ti(OH)_4_ or TiO(OH)_2_ or a mixture of the respective oxide and hydroxide compounds.

The calcinated substrate shows four distinguishable peaks at 636 cm^−^^1^, 515 cm^−^^1^, 397 cm^−^^1^ and 147 cm^−^^1^. These peaks can be assigned to the anatase phase of TiO_2_ [86] and can be clearly distinct from the rutile phase by peak position and relative peak intensity of the peaks [87].

The lower intensive spectral background and the more defined coating composition as well as the more intense spectra in the SERS measurements from Figure 6, show that the calcinated coating is superior to the TTIP substrates dried at RT.

To analyse the changes in wettability of the calcinated TiO_2_ coating, WCA measurements were performed and summarised in Table 1. It becomes clear that the plasma treatment significantly reduced the contact angle of the water droplets on the aluminium substrates and the WCA is even much more reduced on the TiO_2_ layer.

**Figure 8:**
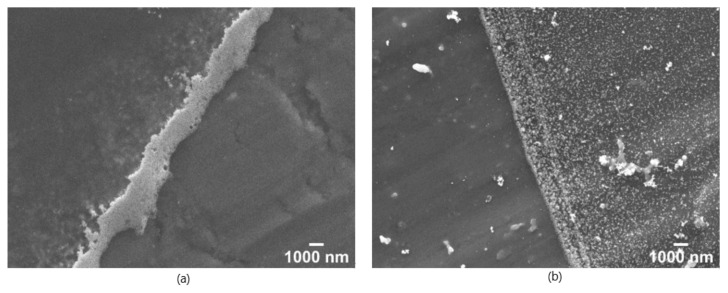
SEM image of the edge region of two SERS substrates at a 5000× magnification with a WD 6 mm and 15 kV acceleration voltage. (**a**) On the left side AuNPs are coated onto the untreated aluminium plates. (**b**) On the right side the AuNPs were coated onto a 4% TTIP coated and calcinated at 600 °C for 5 h plasma treated substrate.

**Figure 9:**
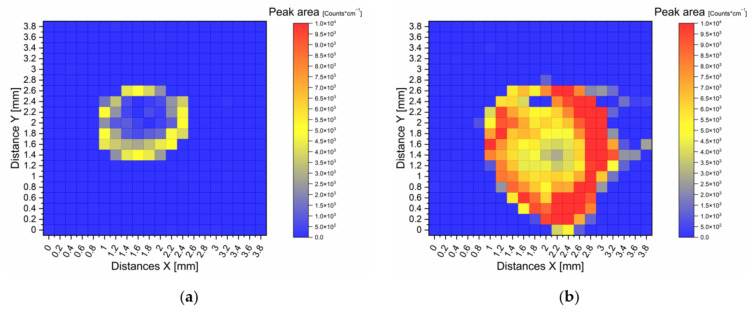
Heat map representations of Raman mappings on two SERS substrates. The peak area of the R6G peak at 1200 cm^−1^ was evaluated. The spectral area within the grey column of Figure 6 was used for integration. The substrates were analysed with a 638 nm laser at 35 mW and 0.96 ng (2 µL of 10^−6^ mol/L) R6G was applied by spiking a 2 µL solution onto the substrates. (**a**) On the left side a SERS substrate with AuNPs on untreated aluminium and (**b**) on the right side AuNPs on sintered TiO_2_ coating.

This reduction in WCA of plasma treated aluminium substrates to respective TiO_2_ calcinated aluminium substrates show the hydrophilization of the surfaces and thereby the major influence on the droplets, which is noticeable in Figure 8. On the one hand, the contact area is significantly increased, which can also be seen in Figure 9, while on the other hand, also the particle transport to the edge area of the droplets is, obviously disturbed, so that the particle distribution should be more uniform and the CRE is reduced.

### 3.3. SERS-Enhancement and the Reduced Coffee Ring Effect

From the edge areas of the droplets as shown in the SEM images in Figure 8, the differences between an untreated (a) and a TTIP coated and calcinated aluminium substrate (b) become clear. The edge of the dried droplet on the untreated aluminium substrate is ~1 µm thick and it consists of a densely packed area of AuNPs. On the TiO_2_ -treated substrate, the actual edge is not that pronounced and the particles are more evenly distributed over a wider area.

Compared to the images in Figure 5, which were taken near the centre of the droplet, the number of gold nanoparticles is increased in the edge area despite the TiO_2_ treatment, but the AuNPs are less strongly fixed at the edge. This indicates that the particle transport within the droplet is not as strong, meaning the CRE is weakened and reduced. Therefore, fewer nanoparticles are transported to the edge and the nanoparticles should be more homogeneously distributed inside the dried droplet. Consequently, these substrates should show a higher SERS enhancement over the entire dried droplet.

To investigate this enhancement, the SERS active areas were again spiked with 2 µL of a 10^−^^6^ M R6G solution, and the respective SERS active area was scanned with a 20 by 20 grid over 4 by 4 mm area. The peak area of the R6G peak at 1200 cm^−^^1^, noticeable in the grey column in Figure 6, was then integrated. For the integration, the range of 1150 cm^−^^1^ to 1250 cm^−^^1^ was isolated from all mapping spectra, followed by a linear baseline correction, before the peak areas were integrated. They were plotted in heat map representations in Figure 9, in which the peak area is calculated from the measurements in the centre of the grid squares. Here, the left mapping (a) shows a SERS active layer of AuNPs on untreated aluminium and the right mapping (b) shows a SERS active layer of AuNPs on calcinated TiO_2_.

On the untreated substrate, a thin edge area with a peak area of 2500 to 5000 Counts cm^−1^ are present in a form of a ring with a diameter of approx. 1–1.2 mm. The TiO_2_ treated SERS substrate shows a clear widening of the entire SERS active area to 2 mm diameter and the peak area is significantly higher. Despite that, the TiO_2_ SERS substrate also has a ring-like shape, the overall intensity of this ring, with more than 10,000 Counts per cm^−^^1^, is much higher compared to the untreated aluminium substrate. Additionally, this edge area of the TiO_2_ substrate is much wider than those of the untreated substrate and the inner area shows higher peak areas compared to the untreated substrate.

As expected, the reduction of surface tension by the hydrophilic layer leads to a larger SERS active surface by reducing the influence of the CRE on the particle transport within the drying process. Because of this, the particles are better distributed within the coated area and the total SERS active area shows a better SERS enhancement. The nanostructured TiO_2_ surface can still be used as a good SERS background, since no intrinsic spectrum was detectable. Furthermore, the better SERS enhancement of the TiO_2_ substrate could also be attributed to enhancement contributions by the TiO_2_ nanostructure, as various publications have reported the enhancement properties by TiO_2_ due to CE through charge transfer interactions [48].

In this work, it was shown that controlled hydrophilic SERS areas by TiO_2_-coatings improve the AuNPs distribution not only on a µm scale but also in a more applicable mm scale. This is a very remarkable result and, moreover, of great interest, as SERS mapping approaches are getting more popular in the last year, for example in the field of food safety [88].

## 4. Conclusions

In summary, a method for the fabrication of AuNP-TiO_2_ based SERS substrates on aluminium plates was developed. For this purpose, nanoparticles were synthesised via a sodium citrate reduction of chloroauric acid. Subsequently, aluminium plates were treated with a specific surface treatment and directly coated with TTIP. After a heating process, a nanostructured TiO_2_ surface was made visible by SEM measurements and the hydrophilic character was confirmed by water contact angle measurements. The hydrophilic surface was then coated with the synthesised gold nanoparticles. SEM analysis showed a clear weakening of the CRE during the drying process and a better distribution of the nanoparticles at the edges of the SERS active areas. SERS measurements of R6G showed an overall improvement of the SERS enhancement and was also confirmed with Raman mappings from R6G over the complete SERS active areas in a mm range. Therefore, the obtained substrates have the potential to be very useful in commercial SERS applications.

## Figures and Tables

**Table 1:** WCA measurements of the different types of substrates.

Treatment	Water Contact Angle [°]
Untreated aluminium	89 ± 6
Plasma treated aluminium	23 ± 8
Plasma treated, calcinated TiO_2_	6 ± 3

## Data Availability

The data presented in this study are available on request from the corresponding author.

## Supplementary Materials

The following are available online at https://www.mdpi.com/article/10.3390/nano12050860/s1, Figure S1: High resolution SEM image of AuNPs onto a SERS-subtrate coated with 4% TTIP and sintered at 600 °C.; Figure S2: High resolution SEM image of a substrate with 4% TTIP and sintered at 600 °C. without AuNPs.
