# Supplementary material for: Development of Gold Nanoparticle-Based SERS Substrates on TiO2-Coating to Reduce the Coffee Ring Effect
Source: Nanomaterials (Basel). 2022 Mar 3;12(5):860. doi: 10.3390/nano12050860 (PMC8912524; doi:10.3390/nano12050860)
Supplement: Supplementary file 1 [file nanomaterials-12-00860-s001.zip › nanomaterials-1608716-supplementary.pdf]

nanomaterials

Supplementary Material

Development of Gold Nanoparticle-Based SERS Substrates on TiO_2-Coating to Reduce the Coffee Ring Effect

René Breuch [1,*], Daniel Klein [1], Cassandra Moers [1], Eleni Siefke [1], Claudia Wickleder [2] and Peter Kaul [1,*]

[1] Institute of Safety and Security Research, University of Applied Sciences Bonn-Rhein-Sieg, von-Liebig-Str. 20, 53359 Rheinbach, Germany; daniel.klein@h-brs.de (D.K.); cassandra.moers@h-brs.de (C.M.); eleni.siefke@h-brs.de (E.S.)

[2] Inorganic Chemistry, Department Chemie and Biologie, Cμ - Center for Micro- and Nanochemistry and (Bio)Technology, Faculty of Science and Technology, University of Siegen, Adolf-Reichwein-Str., 57068 Siegen, Germany, wickleder@chemie.uni-siegen.de

* Correspondence: rene.breuch@h-brs.de (R.B.); peter.kaul@h-brs.de (P.K.)

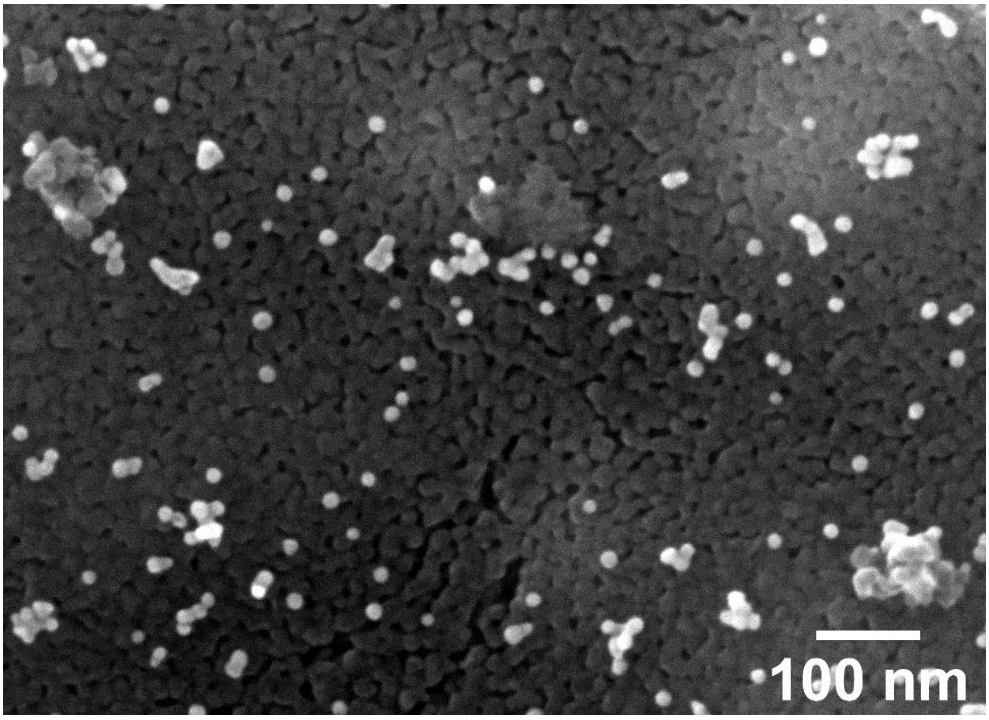

Figure S1. High resolution SEM image of AuNPs onto a SERS-substrate coated with 4% TTIP and sintered at 600 °C a 100,000× magnification with a WD 6 mm and 15 kV acceleration voltage.

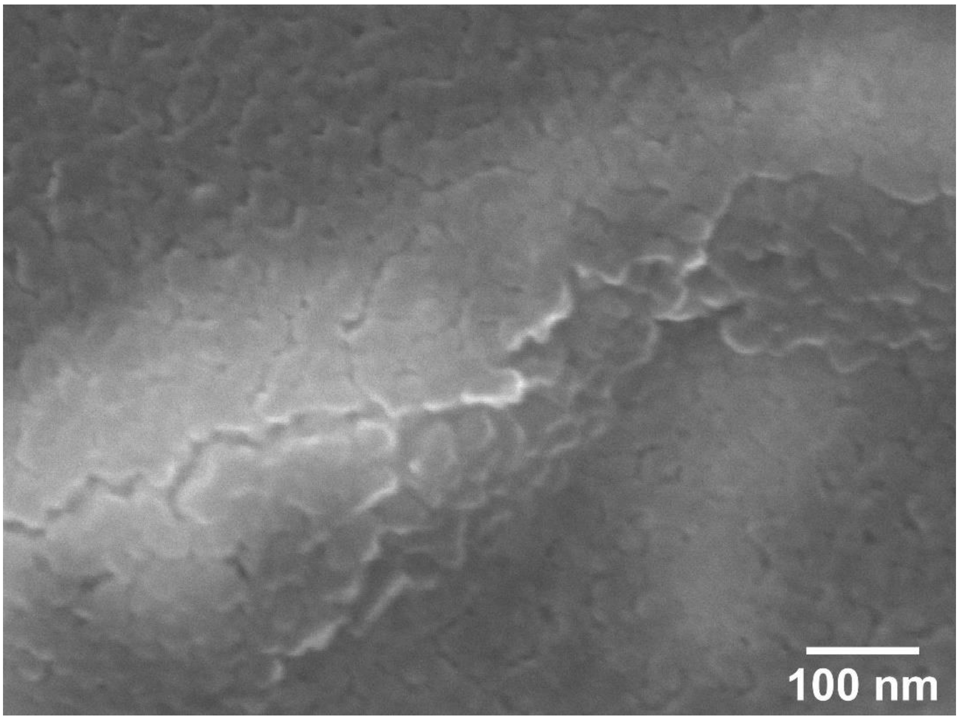

Figure S2. High resolution SEM image of a substrate with 4% TTIP and sintered at 600 °C without AuNPs at 100,000× magnification with a WD 7,5 mm and 15 kV acceleration voltage.
